# Accuracy of machine learning to predict the outcomes of shoulder arthroplasty: a systematic review

**DOI:** 10.1186/s42836-024-00244-4

**Published:** 2024-05-04

**Authors:** Amir H. Karimi, Joshua Langberg, Ajith Malige, Omar Rahman, Joseph A. Abboud, Michael A. Stone

**Affiliations:** 1 Case Western Reserve University School of Medicine, Cleveland, OH 44106 USA; 2 Herbert Wertheim College of Medicine, Miami, FL 33199 USA; 3 Department of Orthopaedic Surgery, Cedars-Sinai Medical Center, Los Angeles, CA 90048 USA; 4 Department of Orthopaedic Surgery, Thomas Jefferson University, Philadelphia, PA 19107 USA

**Keywords:** Machine learning, Shoulder arthroplasty, Artificial intelligence, Patient reported outcomes

## Abstract

**Background:**

Artificial intelligence (AI) uses computer systems to simulate cognitive capacities to accomplish goals like problem-solving and decision-making. Machine learning (ML), a branch of AI, makes algorithms find connections between preset variables, thereby producing prediction models. ML can aid shoulder surgeons in determining which patients may be susceptible to worse outcomes and complications following shoulder arthroplasty (SA) and align patient expectations following SA. However, limited literature is available on ML utilization in total shoulder arthroplasty (TSA) and reverse TSA.

**Methods:**

A systematic literature review in accordance with PRISMA guidelines was performed to identify primary research articles evaluating ML’s ability to predict SA outcomes. With duplicates removed, the initial query yielded 327 articles, and after applying inclusion and exclusion criteria, 12 articles that had at least 1 month follow-up time were included.

**Results:**

ML can predict 30-day postoperative complications with a 90% accuracy, postoperative range of motion with a higher-than-85% accuracy, and clinical improvement in patient-reported outcome measures above minimal clinically important differences with a 93%–99% accuracy. ML can predict length of stay, operative time, discharge disposition, and hospitalization costs.

**Conclusion:**

ML can accurately predict outcomes and complications following SA and healthcare utilization. Outcomes are highly dependent on the type of algorithms used, data input, and features selected for the model.

**Level of Evidence:**

III

## Introduction

Artificial intelligence (AI) utilizes computer systems to simulate cognitive capacities to accomplish goals such as problem-solving and decision-making [1, 2]. A branch of AI known as machine learning (ML) creates algorithms to find connections between preset variables, which are then used to produce prediction models. Algorithms are collections of mathematical processes that explain how variables relate to one another. Algorithms start with data input and work through a set of pre-defined instructions to produce an output [3, 4]. The models are continually improved by using new data, which ultimately refines the prediction ability of the models with little human involvement [5, 6]. There are two types of ML: supervised and unsupervised. Supervised ML is utilized most frequently in healthcare and involves “training” or inputting a dataset of variables, known as *features*, with their relevant outcomes [7]. This allows the computer algorithm to find patterns and associations between features and certain outcomes [7]. After training is completed, the algorithm goes through a “testing” phase where the features of a dataset are applied to the algorithm. The predictions are then compared with known outcomes to determine the algorithm’s accuracy and performance [7]. Unsupervised ML is a data mining method that is used to detect unknown patterns in data without requiring prior human knowledge and intervention [8]. This form of machine learning is typically used more in an exploratory manner without yielding absolute conclusions because the output is highly dependent on whatever parameters are input.

In various prediction problems, ML techniques have demonstrated the ability to outperform conventional approaches such as regression techniques [9, 10]. ML is currently being used more commonly in the field of orthopedic surgery for outcome prediction, diagnostics, and cost-efficiency analyses [3, 11, 12]. ML has been utilized in both total hip and knee arthroplasty to predict patient-reported outcome measures (PROMs) as well as hospital utilization [13–18]. However, there is limited literature available on the utilization of ML in shoulder arthroplasty (SA). The use of anatomic total shoulder arthroplasty (TSA) in the United States has continued to climb due to an aging population as well as expanded indications for reverse total shoulder arthroplasty (rTSA), as seen by a 9.4% yearly increase in procedure volume [19]. Several modifiable and non-modifiable patient characteristics, such as body mass index (BMI), smoking status, or age, increase the risk of complications following SA [20, 21]. Additionally, several studies have shown promise in using ML to predict clinical outcomes such as range of motion (ROM) and PROMs. For instance, Kumar et al., demonstrated ML could predict measures of pain, function, and ROM with an 85 to 94 percent accuracy following TSA [22]. Similarly, Saiki et al., reported that the random forest model algorithm could be useful in predicting knee flexion ROM following TKA [23]. Therefore, the use of ML can aid the shoulder surgeon in determining which patients may be susceptible to complications or poor outcomes following shoulder arthroplasty and can help align patient expectations following TSA and rTSA.

The purpose of this systematic review was to evaluate whether machine learning can be used to predict TSA and rTSA outcomes. Specifically, we asked: (1) Is machine learning able to accurately predict the outcomes and complications after SA? (2) Is machine learning able to accurately predict healthcare utilization including discharge disposition after SA?

## Methods

### Search strategy and criteria

The PubMed, EBSCO host, and Google Scholar electronic databases were searched to identify all studies that evaluated the ability of ML to predict the outcomes of SA. The following keywords were utilized in combination with “AND” or “OR” Boolean operators: (“machine learning” OR “ML” OR “AI” OR “Artificial intelligence” OR “deep learning”) AND (“shoulder arthroplasty” OR “TSA” OR “shoulder surgery” OR “shoulder replacement”).

### Eligibility criteria

For inclusion in this systematic review, each study had to meet the following criteria: (1) articles were currently published, (2) articles reported on the accuracy of ML to predict outcomes of SA, (3) studies were written in the English language. Studies were excluded if they (1) were systematic reviews, (2) were non-peer-reviewed journal publications, case reports, case series, or letters to the editor, (3) provided no relevant outcomes or no outcomes data, (4) were articles that were not given full-text access, (5) or were publications in languages other than English.

### Study selection

**Fig. 1:**
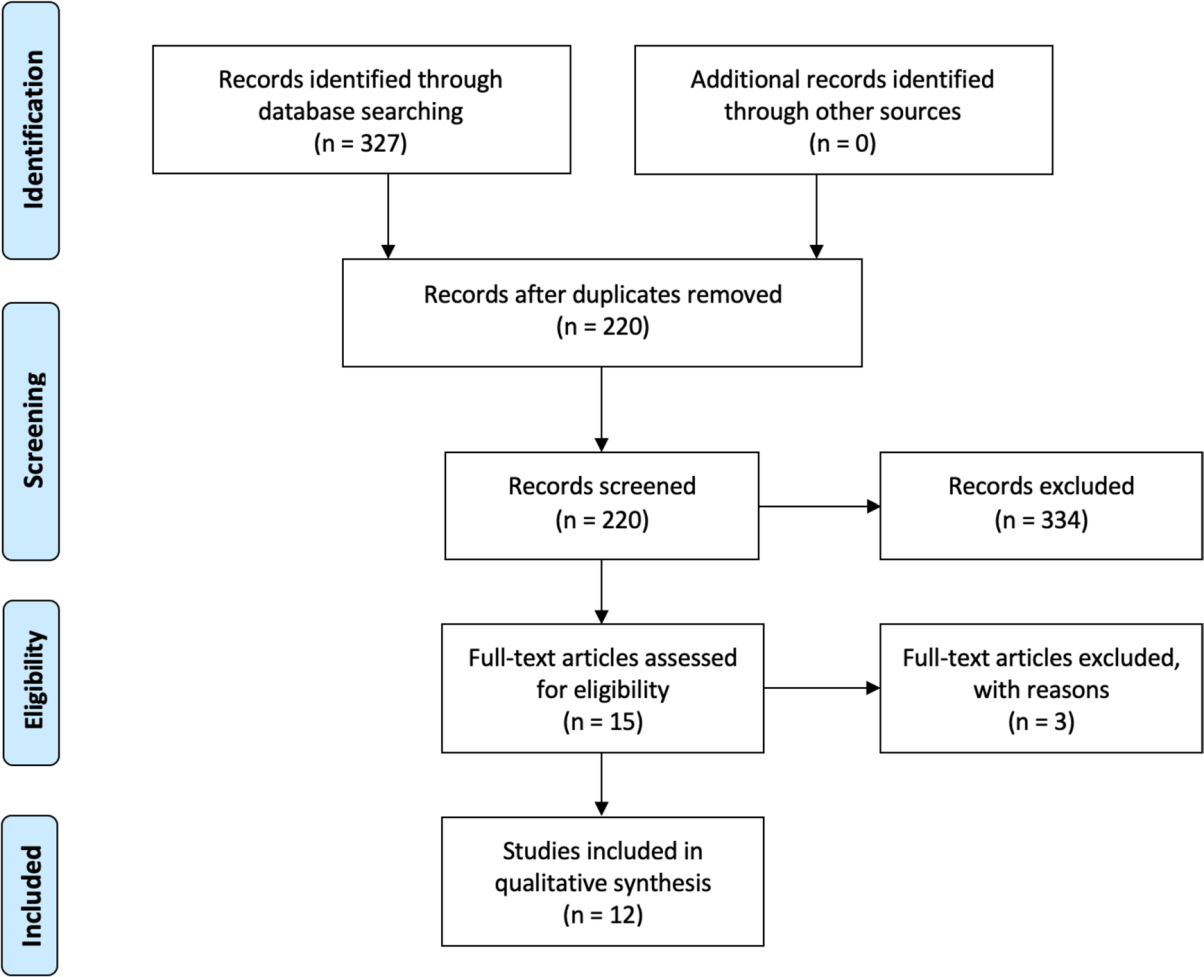
This PRISMA diagram depicts the selection process for article inclusion

In accordance with the Preferred Reporting Items for Systematic Reviews and Meta-Analysis (PRISMA) guidelines, two reviewers (A.K. and J.L.) independently assessed the eligibility of each article to be included in our review [24]. Any differences between the investigators were handled through discussion until a consensus was reached. The initial query yielded 327 publications, which were then screened for appropriate studies that aligned with the purpose of our review. After the removal of duplicates and reading each abstract, 16 studies were selected for further consideration. The full text of each article was then reviewed, of which 12 fulfilled our inclusion and exclusion criteria. A comprehensive examination of each study’s reference list yielded no further papers. Figure 1 depicts the selection procedure.

### Data extraction and collection

A collaborative online spreadsheet (Google Sheets), arranged by two reviewers prior to starting, facilitated data extraction. Two independent reviewers (A.K. and J.L.) extracted the data through a manual full-text review with an identical review strategy. Any disagreements among the investigators were resolved via conversation until consensus was attained. Name of authors, year of publication, study design, sample size, and age (mean), the algorithm used, number of features used, and any relevant outcome reported were extracted from the articles.

### Assessment of methodological quality

The Methodological Index for Non-randomized Studies (MINORS) tool was used by the two reviewers (A.K. and J.L.) to independently evaluate the methodological quality and internal and external validity of all included studies [25]. Twelve evaluation criteria are included in MINORS, of which the first eight are relevant to non-comparative studies with four additional items applicable to comparative studies. A score of 0 (not reported), 1 (reported but inadequate), or 2 is assigned to each item (reported and adequate). For non-comparative studies, the maximum score is 16, and for comparative studies, the maximum score is 24, with higher values indicating higher study quality.

### Data synthesis

A meta-analysis was not carried out due to the heterogeneity of ML algorithms, the presentation of the data, and the outcomes studied. Due to the absence of distinct data, analyses by age groups and gender were also not possible. For each study and result, all the data were gathered and were narratively described.

### Primary and secondary study outcomes

Our primary study goal was to determine the ability of machine learning to predict the outcomes of SA. Of the included studies, nine studies evaluated the accuracy of machine learning to predict SA outcomes. These studies reported PROMs, 30-day complications, and clinical outcomes such as shoulder ROM (with some reporting mean absolute error [MAE]). The secondary objective was to ascertain whether machine learning is capable of forecasting healthcare utilization for SA and the number and type of features that can be used to accurately make predictions. Four studies evaluated either length of stay (LOS), operative time, discharge disposition, or hospitalization costs.

## Results

### Included studies

The final analysis included 12 studies involving 201,649 patients with an average mean age of 65.2 ± 8.23 years (Table 1) [22, 26–36]. There were 43.1% males (86,985) and 56.9% females (114,664). All of the studies were of retrospective design, with an average MINOR score of 14.33 ± 0.78. Five studies used national databases, four of which used the American College of Surgeons National Surgical Quality Improvement Program (ACS-NSQIP); one used the National Inpatient Sample (NIS database); five studies used a multicenter database, and two studies used data from a single institution. Five studies evaluated both rTSA and aTSA separately, while the other six studies did not distinguish between the two. There were 13 different ML algorithms used in the study, including Logistic Regression, K-Nearest Neighbor, Random Forest, Naive-Bayes, Decision Tree, Gradient Boosting Trees, Artificial Neural Network, Linear Regression, XGBoost, Wide and Deep, Stochastic Gradient Boosting, Support Vector Machine, and Elastic-Net Penalized Logistic Regression.

**Table 1:** Characteristics of articles included in final analysis. ACS-NSQIP: American College of Surgeons National Surgical Quality Improvement Program, NIS: National Inpatient Sample, ASES: American Shoulder and Elbow Surgeons score

Title	Author, year	Study design	Database	Sample size (N)	Sex (%male)	Mean age	Machine learning algorithm	Training/test split	Minors
A novel machine learning model developed to assist in patient selection for outpatient total shoulder arthroplasty	Biron et al., (2020) [26]	Retrospective Study	ACS-NSQIP	3,128	44.90%	69.4	Random Forest	70:30	14
Construct validation of machine learning in the prediction of short-term postoperative complications following total shoulder arthroplasty	Gowd et al., (2019) [27]	Retrospective Study	ACS-NSQIP	17,119	56.20%	69.5	-Logistic regression -K-nearest neighbor -Random forest -Naive-Bayes -Decision tree -Gradient boosting trees	80:20	16
The value of artificial neural networks for predicting length of stay, discharge disposition, and inpatient costs after anatomic and reverse shoulder arthroplasty	Karnuta et al., (2020) [28]	Retrospective Study	NIS	111,147	40.80%	69	Artificial Neural Network	70% for training, 10% for validation, 20% for testing	14
What Is the Accuracy of Three Different Machine Learning Techniques to Predict Clinical Outcomes After Shoulder Arthroplasty?	Kumar et al., (2020) [22]	Retrospective Study	MultiCenter	4,782	39.90%	69.6	-Linear regression -XGBoost -Wide and Deep	66.7:33.3	15
Using machine learning to predict clinical outcomes after shoulder arthroplasty with a minimal feature set	Kumar et al., (2021) [31]	Retrospective Study	MultiCenter	5,774	39.30%	70.1	XGBoost	66.7:33.3	14
Use of machine learning to assess the predictive value of 3 commonly used clinical measures to quantify outcomes after total shoulder arthroplasty	Kumar et al., (2021) [32]	Retrospective Study	MultiCenter	2,790	59.10%	N/A	XGBoost	66.7:33.3	15
Using machine learning to predict internal rotation after anatomic and reverse total shoulder arthroplasty	Kumar et al., (2022) [30]	Retrospective Study	MultiCenter	6,468	38.80%	48.7	-Linear regression -XGBoost -Wide and Deep	66.7:33.3	15
Development of a predictive model for a machine learning–derived shoulder arthroplasty clinical outcome score	Kumar et al., (2022) [29]	Retrospective Study	MultiCenter	6,468	38.80%	48.7	-Linear regression -XGBoost -Wide and Deep	66.7:33.3	14
Using machine learning methods to predict nonhome discharge after elective total shoulder arthroplasty	Lopez et al., (2021) [33]	Retrospective Study	ACS-NSQIP	21,544	44.70%	69.1	-Boosted Decision Tree -Artificial Neural Network	80:20	14
Using machine learning methods to predict prolonged operative time in elective total shoulder arthroplasty	Lopez et al., (2022) [34]	Retrospective Study	ACS-NSQIP	21,544	44.70%	69.1	-Boosted Decision Tree -Artificial Neural Network	80:20	14
Machine Learning Can Predict Level of Improvement in Shoulder Arthroplasty	McLendon et al., (2021) [35]	Retrospective Study	Single Institution	472	56%	68	N/A	N/A	14
Development of supervised machine learning algorithms for prediction of satisfaction at 2 years following total shoulder arthroplasty	Polce et al., (2020) [36]	Retrospective Study	Single Institution	413	58.60%	66	-Stochastic gradient boosting -Random forest -Support vector machine -Neural network -Elastic-net penalized logistic regression	80:20	13

*ACS-NSQIP* American College of Surgeons National Surgical Quality Improvement Program, *NIS* National Inpatient Sample, *ASES* American Shoulder and Elbow Surgeons score

### Machine learning and SA outcomes

Nine of the twelve studies agreed that ML could predict the outcomes of SA (Table 2) [22, 27, 29–31, 33–36]. Three studies reported that ML could predict 30-day postoperative complications, with one also advocating for the ability of ML to predict any adverse event, transfusion, extended length of stay, surgical site infection, reoperation, and readmission [27, 33, 34].

**Table 2:** Studies evaluating the ability of machine learning algorithms to predict the outcomes of TSA

Author, year	Outcomes measured	Follow up	Most accurate algorithm	Mean absolute error or accuracy	Outcomes
Gowd et al., (2019) [27]	Postoperative complications	1 Month	N/A	N/A	Machine learning is able to predict postoperative complications in a random sample of a nationwide cohort and outperformed models by comorbidity indices alone utilizing preoperative characteristics
Kumar et al., (2020) [28]	ASES, UCLA, Constant, global shoulder function, VAS pain scores, active abduction, forward flexion, and external rotation	5 + years	Wide and Deep technique	Mean Absolute Error for Wide and Deep Technique: -ASES: ± 10.1 to 11.3 points -UCLA score: ± 2.5 to 3.4 -Constant score: ± 7.3 to 7.9 -Global shoulder function score: ± 1.0 to 1.4 -VAS pain: ± 1.2 to 1.4 -Active abduction: ± 18° to 21° -Forward elevation: ± 15° to 17° -External rotation: ± 10° to 12°	All three machine learning techniques can use preoperative data to predict clinical outcomes at multiple postoperative points after shoulder arthroplasty In addition, the models correctly identified the patients who did and did not experience clinical improvement that exceeded the MCID: 93 to 99 percent accuracy for PROMs and 85 to 94 percent accuracy for measures of pain, function, and range of motion
Kumar et al., (2021) [31]	ASES, Constant score, global shoulder function score, VAS pain scores, active abduction, forward elevation, and external rotation	5 + years	N/A	Mean Absolute Error when using 19 features: -ASES: ± 12 -Constant score: ± 9.8 -Global shoulder function score ± 1.5 -VAS pain: ± 1.4 -Active abduction: ± 21.8° -Forward elevation: ± 19.2° -External rotation: ± 12.6°	Both the full and minimal models exhibited comparable MAEs for predicting each outcome measure at each postoperative time point Additionally, both the full and abbreviated models accurately identified patients who were most at risk of having poor outcomes based on MCID thresholds, enabling risk stratification of patients using only preoperative data (full model accuracy > 82 percent vs. abbreviated model accuracy > 82 percent)
Kumar et al., (2022) [30]	Internal Rotation	5 + years	XGBoost and Wide and Deep	Mean Absolute Error when using 19 features: Wide and Deep: 3–6 months: ± 1.10 6–9 months: ± 1.16 1 year: ± 1.19 2–3 years: ± 1.07 3–5 years: ± 1.04 5 + years: ± 0.96	Active internal rotation following aTSA and rTSA may be precisely predicted at various postoperative time points using a small 19 feature set of preoperative inputs. These predictive algorithms were able to determine which patients will and won't have clinical improvement in their IR score over the MCID (90 percent accuracy for aTSA and 85 percent accuracy for rTSA)
Kumar et al., (2022) [29]	SAS score, ASES score, Constant score	5 + years	Wide and Deep technique	Mean Absolute Error when using 291 features for Wide and Deep Technique: -SAS: ± 7.56 -ASES: ± 10.68 -Constant score: ± 8.25	Although the accuracy of the three machine learning algorithms varied, they all had lower MAE than the baseline average model. Machine learning may be used to predict whether patients will see clinical improvement that is greater than the MCID (96 percent accuracy for both a TSA and rTSA)
Lopez et al., (2021) [33]	Non-home discharge and 30-day postoperative complication rates	1 Month	Both had similar accuracy, but the artificial Neural Network had better discriminative ability	Accuracy for Artificial Neural Network 30-day Postoperative Complication rate Accuracy: -Boosted Decision Tree: 95.5% -Artificial Neural Network: 92.5%	Both Boosted decision tree model and Artificial Neural Networks has a greater than 90% accuracy in predicting 30-day postoperative complications
Lopez et al., (2022) [34]	Prolonged operative time and 30-day postoperative complication rates	1 Month	Artificial Neural Network	Accuracy for Artificial Neural Network 30-day Postoperative Complication rate Accuracy: -Boosted Decision Tree: 95.5% -Artificial Neural Network: 92.5%	Both Boosted decision tree model and Artificial Neural Networks has a greater than 90% accuracy in predicting 30-day postoperative complications
McLendon et al., (2021) [35]	ASES	2 years	N/A	Accuracy of predicting different improvement levels for model 1: - ≤ 28 points: 94% -29 to 55 points: 95% - > 55 points: 94%	Machine learning can reliably predict the extent of improvement following glenohumeral OA shoulder arthroplasty
Polce et al., (2020) [36]	Patient satisfaction	2 years	Support vector machine	N/A	The Support vector machine model demonstrated excellent discrimination and adequate calibration for predicting satisfaction following TSA

*TSA* Total Shoulder Arthroplasty, *ASES* American Shoulder and Elbow Surgeons score, *UCLA* University of California, Los Angeles Score, *VAS* Visual Analog Scale, *MCID* Minimal Clinically Important Differences

ML was also able to predict ROM at different postoperative time points. Kumar et al., in two different studies, reported that machine learning (Wide and Deep and XG Boost) could predict postoperative ROM, with a mean absolute error (MAE) between ± 18° to 21.8° for active abduction, ± 15° to 19.2° for forward flexion and ± 10° to 12.6° for external rotation [22, 31]. Both studies ran independent models for TSA and rTSA cases as well, finding similar predictability between both. Similarly, in a different study, Kumar et al. showed that machine learning could predict postoperative minimal clinically important difference (MCID) internal rotation with a 90% accuracy for anatomic TSA and an 85% accuracy for rTSA [30]. Five articles demonstrated that ML could accurately predict PROMs [22, 29, 31, 35, 36]. Kumar et al. were able to identify patients undergoing either TSA or rTSA that would have PROM improvement exceeding the MCID in multiple studies [22, 29, 31], while McLendon et al. demonstrated that ML could predict the degree of improvement in ASES scores by around 95% [35].

### Machine learning and healthcare utilization for SA

The four studies on LOS, operative time, discharge disposition, or hospitalization costs were in agreement regarding ML ability to predict different aspects of healthcare (Table 3) [26, 28, 33, 34]. Two studies reported that ML could accurately predict LOS of patients, with one study reporting accurate disposition for patients remaining hospitalized ≤ 1 day or > 3 days following SA [26]. Karnuta et al. were able to predict length of stay with an accuracy of 79.1% for acute or traumatic conditions for their inpatient admission and 91.8% for chronic or degenerative conditions [28]. Lopez et al. were able to predict operative time with 85% accuracy [34]. The authors also used two different ML algorithms to predict non-home discharge with an accuracy greater than 90% [33]. Using a different algorithm, Karnuta et al. were able to predict disposition to home with an accuracy of 70% [28]. They also predicted total inpatient costs after SA with an accuracy of 70.3% for acute conditions and 76.5% for chronic conditions [26].

**Table 3:** Studies evaluating the ability of machine learning algorithms to predict healthcare utilization of  TSA

Author, year	Outcomes measured	Follow up	Most accurate algorithm	Mean absolute error	Outcomes
Biron et al., (2020) [26]	Length of stay	N/A	N/A	N/A	Machine learning may be used to predict whether individuals had a one-day LOS or shorter following TSA
Karnuta et al., (2020) [28]	Length of stay, discharge disposition, and inpatient charges	1 Month	N/A	Accuracy in Chronic/degenerative conditions -Total Cost: 76.5% -Length of Stay: 91.8% -Disposition (home): 73.1% Accuracy in Acute/traumatic conditions -Total Cost: 70.3% -Length of Stay: 79.1% -Disposition (home): 72%	For both chronic/degenerative and acute/traumatic shoulder arthroplasty, artificial neural networks displayed medium to high accuracy and reliability in predicting inpatient cost, LOS, and discharge disposition
Lopez et al., (2021) [33]	Non-home discharge and 30-day postoperative complication rates	1 Month	Both had similar accuracy, but the artificial Neural Network had better discriminative ability	Accuracy for Artificial Neural Network Non-home discharge Accuracy: -Boosted Decision Tree: 90.3% -Artificial Neural Network: 89.9%	Machine learning has the capacity to reliably predict non-home discharge following elective TSA
Lopez et al., (2022) [34]	Prolonged operative time and 30-day postoperative complication rates	1 Month	Artificial Neural Network	Accuracy for Artificial Neural Network Prolonged operative time Accuracy: -Boosted Decision Tree: 85.6% -Artificial Neural Network: 84.7%	Machine learning models can predict which patients are more likely to require longer TSA operations

### Machine learning and features

Six of the articles reported on the number and type of features required for ML to make any of the above predictions (Table 4) [29–32, 35, 36]. In three different studies, Kumar et al. demonstrated that utilizing the minimal-feature model (19 features) had comparable accuracy as compared to using the full-feature model (291 features) in predicting ROM and PROMs for either TSA or rTSA (Table 5) [29–31]. Additionally, they discovered that only slight improvements in MAEs were observed for each outcome measure when the minimal model was supplemented with information on implant size and/or type as well as measurements of native glenoid anatomy [29, 30]. In all of their studies, Kumar et al. showed that the presence of radiographical information does not provide significant predictive ability to ML algorithms [29–31]. Follow-up duration and composite ROM were the most important or predictive features for the full-feature model and the minimal-feature model, respectively [27, 29].

**Table 4:** Studies evaluating the type and number of features required for machine learning algorithms to predict the outcomes of TSA

Author, year	Number of features	Type of features	Outcome measure	Outcomes
Kumar et al., (2021) [31]	19 and 291	-Full feature group (291) includes: Demographic data, diagnoses, comorbidities, implant type, preoperative ROM, preoperative radiographic findings, and preoperative PROMs (ASES, SPADI, SST, UCLA, and Constant metrics) -Minimal feature group (19) includes: Age, weight, height, sex, Previous shoulder surgery, surgery on dominant arm, diagnosis, comorbidities, ROM, Global Shoulder Function score, VAS score, Pain at worse, pain when lying on the side, pain when touching back of neck, and pain when pushing with effective arm	ASES, Constant score, global shoulder function score, VAS pain scores, active abduction, forward elevation, and external rotation	A comparison of MAEs for the full and minimal models shows that each model had comparable prediction accuracy for each outcome measure. When the minimal model was augmented with data on implant size and/or type, as well as measurements of native glenoid anatomy, only minor improvements in MAEs were seen for each outcome measure
Kumar et al., (2021) [32]	291	Demographic data, diagnoses, comorbidities, implant type, preoperative ROM, preoperative radiographic findings, and preoperative PROMs (ASES, SPADI, SST, UCLA, and Constant metrics)	N/A	The predictive value of the questions in the UCLA score exceeded that of the Constant questions, while the Constant questions were more predictive than the ASES questions. In addition, the preoperative SPADI score was more predictive than the preoperative ASES, Constant, and UCLA scores. Ultimately, we determined that subjective self-assessments of pain, as well as objective measurements of active range of motion and strength, were the most predictive types of preoperative input questions
Kumar et al., (2022) [29]	19 and 291	-Full feature group (291) includes: Demographic data, diagnoses, comorbidities, implant type, preoperative ROM, preoperative radiographic findings, and preoperative PROMs (ASES, SPADI, SST, UCLA, and Constant metrics) -Minimal feature group (19) includes: Age, weight, height, sex, Previous shoulder surgery, surgery on dominant arm, diagnosis, comorbidities, ROM, Global Shoulder Function score, VAS score, Pain at worse, pain when lying on the side, pain when touching back of neck, and pain when pushing with effective arm -Minimal feature set + implant data includes: All the features from the Minimal feature group plus implant size/type data, and computed tomographic and radiographic-based measurements of native glenoid version and inclination	Internal Rotation	At each prediction time point, the degree of prediction accuracy across the three major model input categories was comparable between the full feature model and the minimal feature with and without implant/imaging data The preoperative composite ROM score was found as the most relevant feature driving each minimal feature set, whereas the follow-up length was the most meaningful factor driving the Full feature group, with composite ROM score being the third most important feature
Kumar et al., (2022) [30]	19 and 291	-Full feature group (291) includes: Demographic data, diagnoses, comorbidities, implant type, preoperative ROM, preoperative radiographic findings, and preoperative PROMs (ASES, SPADI, SST, UCLA, and Constant metrics) -Minimal feature group (19) includes: Age, weight, height, sex, Previous shoulder surgery, surgery on dominant arm, diagnosis, comorbidities, ROM, Global Shoulder Function score, VAS score, Pain at worse, pain when lying on the side, pain when touching back of neck, and pain when pushing with effective arm -Minimal feature set + implant data includes: All the features from the Minimal feature group plus implant size/type data, and computed tomographic and radiographic-based measurements of native glenoid version and inclination	SAS score, ASES score, Constant score	SAS score was the most predictive/accurate variable to predict aTSA and rTSA outcomes for all 3 machine learning techniques followed by the Constant score and finally the ASES score. For all the outcomes, follow-up duration was the most important feature for the Full feature group, while composite ROM was the most important feature for the minimal feature group
McLendon et al., (2021) [35]	N/A	-Model 1: using of all baseline variables -Model 2: omitting morphological variables -Model 3: omitting ASES variables	ASES	Latent factors and morphological variables had most accurate predictions when combined which suggests that both structural pathology and patient perceptions are important for achieving the best results/predictions
Polce et al., (2020) [36]	16	Age, BMI, sex, insurance status, preoperative duration of shoulder-related symptoms > 2 years (yes or no), smoking status, history of ipsilateral shoulder surgery, diabetes mellitus or HTN, preoperative physical activity, humeral component fit, diagnosis, procedure (aTSA or rTSA), ASES, SANE, and subjective Constant-Murley score	Patient satisfaction	Age, insurance status, smoking status, BMI, diabetes mellitus, preoperative activity, preoperative duration of symptoms, diagnosis, procedure, and baseline SANE score were the 10 predictive factors revealed by RFE and cross-validation during model training The baseline SANE score, exercise and activity, insurance status, diagnosis, and preoperative duration of symptoms were the five most predictive variables that went into the SVM model when they were averaged across all patients

*TSA* Total Shoulder Arthroplasty, *ASES* American Shoulder and Elbow Surgeons score, *UCLA* University of California, Los Angeles Score, *VAS* Visual Analog Scale, *MCID* Minimal Clinically Important Differences, *SANE* Single Assessment Numeric Evaluation, *SAS* Shoulder Arthroplasty Smart

**Table 5:** The 19 features included in the minimal-feature model by Kamath et al.

Features	Description
Age	Age in years
Weight	Weight in lbs
Height	Height in inches
Sex	Male or female
Prior shoulder surgery	Has the patient previously had a surgical operation on the shoulder?
Dominant-sided surgery	Will the upcoming arthroplasty be on the patient’s dominant shoulder?
Primary diagnosis	What is the patient’s primary diagnosis?
Comorbidities	What are the patients’ comorbidities?
Preop active abduction	Active arm elevation in frontal plane
Preop active FE	Active arm elevation in sagittal plane,
Preop active ER	Active lateral rotation of arm, with arm at side
Preop passive ER	Passive lateral rotation of arm, with arm at side
Preop IR score	Active medial rotation of arm, with arm at side; unitless: 8-point numeric scale with the following discreet assignments based on motion to vertebral segments: 0, no motion; 1, hip; 2, buttocks; 3, sacrum; 4, L5 to L4; 5, L3 to L1; 6, T12 to T8; and 7, T7 or higher
Preop Global function score	Patient assessment of ability to use shoulder prior to surgery via Global Shoulder Function score; 11-point score (0–10), with 10 indicating full or normal mobility
Preop VAS	Patient assessment of pain experienced on daily basis prior to surgery via VAS pain score; 11-point score (0–10), with 10 indicating extreme pain
Preop pain at worst	Patient assessment of worst pain experienced on daily basis prior to surgery; 11-point score (0–10), with 10 indicating extreme pain
Preop pain lying on the side	Patient assessment of pain experienced when lying on affected side prior to surgery; 11-point score (0–10), with 10 indicating extreme pain
Preoperative pain when touching back of neck	Patient assessment of pain experienced when touching back of neck prior to surgery; 11-point score (0–10), with 10 indicating extreme pain
Preoperative pain when pushing with affected arm	Patient assessment of pain experienced when pushing with affected arm prior to surgery; 11-point score (0–10), with 10 indicating extreme pain

*lbs* pounds, *VAS* visual analogue scale, *L* Lumbar, *T* Thoracic, *FE* forward elevation, *ER* external rotation, *IR* internal rotation

Polce et al. were able to accurately predict patient satisfaction based on 16 features. For the support vector machine algorithm, they found the five most predictive variables to predict patient satisfaction were baseline SANE score, exercise and activity, insurance status, diagnosis, and preoperative duration of symptoms [36]. In two different studies, Kumar et al. reported on the best predictors of postoperative outcomes, citing preoperative Shoulder Pain and Disability Index (SPADI) scores, postoperative SAS scores, ASES, UCLA, and Constant scores overall as the most predictive [29, 32]. Finally, McLendon et al. demonstrated that both the preoperative ASES and morphological variables of the shoulder were required in combination to accurately predict the improvement in ASES scores [35].

## Discussion

All 12 articles were consistent in reporting that machine learning could accurately predict outcomes and complications after SA. ML also seems to be successful at predicting post-SA PROMs. While ASES was the most common outcome score predicted, there was a high variability in outcomes tested and predicted among studies. Multiple studies also focused on predicting improvement greater than established PROM MCIDs [22, 29, 35]. This level allows for increased standardization and clinical conclusions from the data and should be used in future studies as well.

Lopez et al. and Gowd et al. both validated the ability of their ML algorithms to predict complications, while Gowd et al. also noted that their algorithm outperformed comorbidity indices-alone models [27, 33, 34]. This is similar to results seen in both hip and knee arthroplasty. For instance, Harris et al. demonstrated that neural network models had good accuracy in determining the likelihood a patient would experience renal or cardiac complications [15]. The ability of ML to predict outcomes can help with surgical risk classification and enable surgeons to use measures to lower complications and improve outcomes.

In addition to outcome prediction following SA, ML was able to predict different healthcare utilization factors such as LOS and discharge disposition with high accuracy and reliability. This is a valuable tool that may help lower healthcare-related costs. Calkins et al. reported that outpatient SA led to a charge reduction of $25,509 to $53,202 per patient compared to inpatient SA, and this data can be used preoperatively for patient disposition planning [37]. Additionally, disposition planning to non-home facilities is commonly delayed, resulting in extended hospital LOS, higher expenses, and increased patient morbidity and mortality [38–40]. By using ML to predict which patients would be discharged to non-home facilities, surgeons may organize ahead of time to accelerate the discharge process, which may lower healthcare-related costs and potentially mitigate adverse events.

Although ML in clinical use is promising, the accuracy of prediction is highly sensitive to the algorithm used and the number and type of features chosen as input values. Kumar et al. were able to demonstrate accurate PROMs following SA using as little as 19 features [29–31]. The authors found that the SAS score, which is a composite of ASES sub-questions, was one of the most accurate features. Unfortunately, there is no consensus on the type or amount of features that most accurately predict outcomes among a wide variety of patients. There were 13 different algorithms used across studies, all of them showing relatively strong predictive ability. While increasing features logically seems to add granularity and detail to predictive algorithms, it also adds an element of complexity that may not be easily reproducible or clinically significant. As more algorithms are created and validated, the most efficient and generalizable algorithm will hopefully be elucidated. However, currently, there does not seem to be a specific algorithm that is significantly superior to other types of algorithms. In our study, seven articles utilized multiple algorithms for their studies and demonstrated similar accuracy between the algorithms used.

In addition, only four studies ran independent models for TSA and rTSA cases [22, 29–31]. Karnuta et al. was the only other study that separated TSA and rTSA cases [28]. The other studies either pool all cases together or do not differentiate which types of shoulder replacements they use. Furthermore, there is some inconsistency among the included articles about how shoulder arthroplasty is referred to (TSA denoting all shoulder arthroplasties versus denoting only anatomic total shoulder arthroplasties). Having a clear delineation of which procedures are being included as well as separate models for TSA and rTSA cases is important for all future ML studies to do. The two procedures, including technical factors as well as patient selection, are very different. Factors that lead to successful outcomes are also very different in both procedures, highlighting the need for independent modeling. Even though the limited available studies had similar predictability for all modeled outcomes for both TSA and rTSA models, this needs to be further studied (and statistically compared, which was not done in our review) to definitively determine whether one model can accurately predict both types of procedures as one cohort.

Finally, many studies only tested their algorithms at one center with one patient population. Testing their algorithms among multiple centers and patient populations strengthens the algorithm’s ability to accurately predict outcomes in a wider variety of populations, increasing its generalizability to all patient types. Furthermore, all 12 studies were internally validated. It is also important to externally validate these algorithms, given the propensity for ML algorithms to over-fit data that it has been exposed to and under-fit data it has not yet been exposed to. External validations will help increase trust and adoption of these new tools. However, these points highlight the importance of further testing of ML algorithms to not only determine a universal algorithm that is used consistently across the country but also to determine the set of features that allows for accurate predictions using differing algorithms. In a systematic review of the availability of externally validated ML models with orthopedic outcomes, Groot et al. reported that only 10/50 of the ML models predicting orthopedic surgical outcomes were externally validated, but those that had good discrimination ability [41]. Despite the crucial need to evaluate prediction models on new datasets, this is seldom done due to data protection by institutions and journal preferences for publishing developmental studies. Algorithms with poor external validation performance may face publication bias.

### Limitations

Our analysis has several limitations. Firstly, all the included studies in our analysis had a retrospective design, which limits the capability to accurately determine the ability of machine learning to predict outcomes of SA prospectively. Secondly, there was heterogeneity across the studies regarding the type of algorithms used and the number of features used to train the algorithm, and the outcomes they studied. However, this may allow for improved generalizability of our results as there are frequently incomplete patient data depending on the algorithm used. Thirdly, five of the studies included were by Kumar et al., which limits the generalizability of the study. However, they used a multicenter database, which contained a large composite of patient information from multiple institutions, thus increasing the generalizability of the study. Despite these limitations, our systematic review provides the first summary of the available literature on the ability of machine learning to predict the outcomes of shoulder arthroplasty and healthcare utilization.

## Conclusion

Our systematic review found that machine learning could accurately predict both ROM and PROMs, complications, and healthcare utilization of patients undergoing TSA and rTSA. These findings encourage continued efforts to utilize both machine learning and other technology to improve patient outcomes of shoulder arthroplasty. Efforts should focus on determining which patients are at risk of poor outcomes following shoulder arthroplasty and potential ways to mitigate these risks preoperatively and provide the patient with appropriate preoperative counseling to enhance shared decision-making. With multiple machine learning algorithms being utilized in the current literature, future studies should establish a consistent algorithm to ensure patients who are at an increased risk for complication are reliably identified to receive optimal treatment.

## Data Availability

Not applicable.
